# *N*-acetylcysteine regulates dental follicle stem cell osteogenesis and alveolar bone repair via ROS scavenging

**DOI:** 10.1186/s13287-022-03161-y

**Published:** 2022-09-08

**Authors:** Zhaosong Meng, Jiacheng Liu, Zhipeng Feng, Shuling Guo, Mingzhe Wang, Zheng Wang, Zhe Li, Hongjie Li, Lei Sui

**Affiliations:** 1 Department of Oral and Maxillofacial Surgery, School and Hospital of Stomatology, Tianjin Medical University, Tianjin, China; 2 Department of Prosthodontics, School and Hospital of Stomatology, Tianjin Medical University, 12 Qixiangtai Road, Tianjin, 300070 China; 3 School of Stomatology, Tianjin Medical University, Tianjin, China

**Keywords:** *N*-acetylcysteine, Dental follicle stem cells, Cell differentiation, Bone development, Reactive oxygen species, Signal transduction

## Abstract

**Background:**

Dental follicle stem cells (DFSCs) show mesenchymal stem cell properties with the potential for alveolar bone regeneration. Stem cell properties can be impaired by reactive oxygen species (ROS), prompting us to examine the importance of scavenging ROS for stem cell-based tissue regeneration. This study aimed to investigate the effect and mechanism of *N*-acetylcysteine (NAC), a promising antioxidant, on the properties of DFSCs and DFSC-based alveolar bone regeneration.

**Methods:**

DFSCs were cultured in media supplemented with different concentrations of NAC (0–10 mM). Cytologic experiments, RNA-sequencing and antioxidant assays were performed in vitro in human DFSCs (hDFSCs). Rat maxillary first molar extraction models were constructed, histological and radiological examinations were performed at day 7 post-surgery to investigate alveolar bone regeneration in tooth extraction sockets after local transplantation of NAC, rat DFSCs (rDFSCs) or NAC-treated rDFSCs.

**Results:**

5 mM NAC-treated hDFSCs exhibited better proliferation, less senescent rate, higher stem cell-specific marker and immune-related factor expression with the strongest osteogenic differentiation; other concentrations were also beneficial for maintaining stem cell properties. RNA-sequencing identified 803 differentially expressed genes between hDFSCs with and without 5 mM NAC. “Developmental process (GO:0032502)” was prominent, bioinformatic analysis of 394 involved genes revealed functional and pathway enrichment of ossification and PI3K/AKT pathway, respectively. Furthermore, after NAC treatment, the reduction of ROS levels (ROS, superoxide, hydrogen peroxide), the induction of antioxidant levels (glutathione, catalase, superoxide dismutase), the upregulation of PI3K/AKT signaling (PI3K-p110, PI3K-p85, AKT, phosphorylated-PI3K-p85, phosphorylated-AKT) and the rebound of ROS level upon PI3K/AKT inhibition were showed. Local transplantation of NAC, rDFSCs or NAC-treated rDFSCs was safe and promoted oral socket bone formation after tooth extraction, with application of NAC-treated rDFSCs possessing the best effect.

**Conclusions:**

The proper concentration of NAC enhances DFSC properties, especially osteogenesis, via PI3K/AKT/ROS signaling, and offers clinical potential for stem cell-based alveolar bone regeneration.

**Supplementary Information:**

The online version contains supplementary material available at 10.1186/s13287-022-03161-y.

## Background

Stem cells derived from human dental tissues with properties of self-renewal, multilineage differentiation and immunomodulation have been intensively investigated for stem cell-based regenerative medicine [1, 2]. Compared to other dental stem cells, dental follicle stem cells (DFSCs) are mainly isolated from human dental follicles within the developing wisdom teeth germ, so their plasticity is much better with the advantages of easier clinical access and less ethical controversy [3, 4]. DFSCs are differentiated naturally into osteoblasts and cementoblasts; therefore, they are a viable candidate for tissue engineering and regenerative medicine, particularly in alveolar bone [5, 6].

Stem cell properties rely on various intrinsic and extrinsic factors [7–9]. Accumulative reactive oxygen species (ROS) produced from endogenous and exogenous sources accompany decline in oxidative defense to cause cellular oxidative stress, impair stem cell properties and significantly reduce the efficacy of stem cells [10–12]. Therefore, scavenging accumulative ROS is a beneficial strategy for the preservation of stem cell properties and the feasibility of stem cell-based therapy [13, 14].

A sophisticated antioxidant system comprising of endogenous and exogenous antioxidants is required for maintaining cellular redox homeostasis and functions. Glutathione (GSH) is the most important endogenous antioxidant and supplementation of exogenous GSH has been proven to preserve stem cell properties by inhibiting ROS generation in vitro [13, 15]. Whereas, GSH administration is not favorable in vivo due to its rapid degradation or oxidation [16, 17]. Since GSH cannot be administered directly, the creative notion of precursors has been proposed to mimic physiologic and pharmacologic effects of GSH.

*N*-acetylcysteine (NAC) which has a proven role in specific clinical settings is a precursor for GSH synthesis and is considered as a modulator of the intracellular redox state [18]. NAC is an amino acid derivative with small molecular weight presented in healthy cells at millimolar concentrations and has strong antioxidant capacity. NAC is not only rapidly deacetylated to promote intracellular GSH synthesis, but also eliminates electrophilic groups of free radicals directly through free thiol side-chain [19] (Additional file 1: Fig. S1A). Recent studies have highlighted a significant role of NAC in preserving the properties of stem cells through antioxidative effects, including bone marrow stem cells [20, 21] and adipose-derived stem cells [22]. Furthermore, the synergistic effect of NAC and stem cells has been proved to yield superior therapeutic efficacy than monotherapy in bone injury [20], preclinical interstitial cystitis [23] and bioroot engineering [24]. NAC is a life-saving drug with low toxicity and few side effects, which greatly facilitates its clinical research. Relationship between overdose and therapy seems to be the only apparent limitation in its efficacy [18]. Moreover, accumulating evidence indicates that ROS-dependent signaling pathways play a crucial role in bio-modulation effects of exogenous antioxidants [25–27]. Unfortunately, a comprehensive overview of physiological and pharmacological evidence of NAC on dental stem cells is insufficient. Nor is the underlying mechanism known.

The current study aimed to study the effects of NAC on the properties of DFSCs, explore possible molecular mechanisms utilizing RNA sequencing and investigate its use as a potential therapeutic agent for dental stem cell-based alveolar bone augmentation using a rodent animal model.

## Methods

### NAC preparation

NAC powder (Aladdin Chemical, Shanghai, China) was dissolved in HEPES buffer (Solarbio, Beijing, China) for an NAC stock solution (500 mM stock, pH = 7.0) stored at 4℃ and protected from light.

### Human DFSC isolation and culture

The sample contained 5 fresh human dental follicles from 5 individuals who were selected to volunteer in this study according to the inclusion criteria: age between 13 and 22 years, healthy subjects, with good oral health conditions, referred for third mandibular molar extraction due to orthodontic or embed-impacted reasons in Tianjin Medical University Stomatology Hospital, unerupted teeth with low density of the dental follicle tissues around crowns observed on panoramic radiographs (Additional file 1: Fig. S1B and C). For this purpose, approval from the Ethics Committee of the Affiliated Stomatological Hospital of Tianjin Medical University (permission no. TMUSH-hMEC2016082) and written consent from each patient were obtained. Isolation of human DFSCs (hDFSCs) was performed according to our previous study [28] and characterization is shown in Additional file 2: Fig. S2. Cells were cultured in minimum essential medium-α (α-MEM; HAKATA, Shanghai, China) containing 10% heat-inactivated fetal bovine serum (FBS; HAKATA, Shanghai, China) and 1% penicillin/streptomycin (Solarbio, Beijing, China) in a humidified atmosphere of 5% CO_2_ at 37 °C. When adherent cells reached approximately 80% confluent, they were washed with phosphate buffer solution (PBS; Solarbio, Beijing, China), detached using 0.25% trypsin with 1 mM EDTA-4Na (Gibco, MA, USA) and seeded for passage culture. For NAC treatment, each culture medium supplemented with or without NAC stock solutions (final concentrations of 0, 2.5, 5, or 10 mM) was added immediately after seeding and renewed every 2 days. For PI3K inhibition, cells were treated with LY294002 (10 µM; Beyotime, Shanghai, China) for 2 days. Cells from passage 6–12 were used for all experiments. This study was conducted according to World Medical Association Declaration of Helsinki, ethical principles for medical research involving human subjects.

### Oxidant and antioxidant assays

hDFSCs were cultured as above for 2–4 days before assays. According to a ROS assay kit (Beyotime, Shanghai, China), after washed three times with PBS, hDFSCs were incubated in 10 µM DCFH-DA solution at 37 °C for 20 min and observed by a laser confocal microscope (Zeiss, Oberkochen, Germany) at the wavelength of 488/525 (excitation/emission). For quantitation, hDFSCs were dissociated by 0.25% trypsin–EDTA and incubated as described above and the relative mean fluorescence intensity (MFI) was analyzed and normalized to the control using flow cytometry (BD, NY, USA) and FlowJo (BD, NY, USA). A superoxide assay kit (Beyotime, Shanghai, China), a hydrogen peroxide (H_2_O_2_) assay kit (Solarbio, Beijing, China), a total glutathione assay kit (Beyotime, Shanghai, China), a total superoxide dismutase (SOD) assay kit (Beyotime, Shanghai, China) and a catalase (CAT) assay kit (Beyotime, Shanghai, China) were used, respectively. Absorbance was measured by a microplate reader (Tecan, Männedorf, Switzerland). Protein concentration of each sample was determined at A280 using Nanodrop2000 (Thermo Scientific, MA, USA).

### Cell morphology assays

hDFSCs were incubated with NAC and observed at defined end points (day 1, 2 and 3) using an inverted microscope (Zeiss, Oberkochen, Germany). hDFSCs incubated with NAC for 3 days were seeded on autoclaved coverslips overnight. According to the manufacturer’s instructions, cells were stained with FITC-phalloidin (Solarbio, Beijing, China) and nuclei of cells were stained by DAPI (Vector, Burlingame, CA). Images were acquired with a laser confocal microscope (Zeiss, Oberkochen, Germany).

### Oil red o staining

The adipogenic-inducing medium was α-MEM supplemented with 10% FBS, 1% penicillin/streptomycin, 111 μg/mL IBMX (Solarbio, Beijing, China), 72 μg/mL indomethacin (Solarbio, Beijing, China), 5 μg/mL insulin (Aladdin Chemical, Shanghai, China) and 0.4 μg/mL dexamethason (Sigma-Aldrich, MO, USA). Oil red o staining after 15-day induction was carried out using a modified oil red o staining kit (Beyotime, Shanghai, China).

### Immunofluorescent staining

hDFSCs and rDFSCs at passage 3 were grown on autoclaved coverslips. At room temperature, after fixed with 4% paraformaldehyde (Solarbio, Beijing, China) for 15 min and washed three times with PBS, cells were permeabilized for 10 min with 0.2% Triton X-100 solution (Solarbio, Beijing, China), blocked in 1% bull serum albumin (Solarbio, Beijing, China) solution for 1 h and incubated with appropriate primary antibodies for 3 h and secondary antibodies for 1 h in the dark. Antibodies are listed in Additional file 6: Table S1. Nuclei of cells were stained by DAPI (Vector, Burlingame, CA) for 10 min in the dark. Images were acquired with a laser confocal microscope (Zeiss, Oberkochen, Germany).

### Cell viability assays

hDFSCs were seeded immediately at a density of 4 × 10^3^/well in a 96-well plate and incubated with NAC at 37 °C and 5% CO_2_. At defined end points (24, 48, 72 h), cells were rinsed with PBS three times to remove residual NAC and cell viability was quantified using a cell counting kit-8 (CCK-8; Solarbio, Beijing, China) following the manufacture’s protocol for 90 min. A microplate reader (Tecan, Männedorf, Switzerland) was used to measure absorbance at 450 nm.

### Osteo-differentiation analysis

The osteogenic-inducing medium was α-MEM supplemented with 10% FBS, 1% penicillin/streptomycin, varying concentrations of NAC, 10 mM Na-β-glycerophosphate (Solarbio, Beijing, China), 100 nM dexamethason (Sigma-Aldrich, MO, USA) and 50 μg/mL vitamin C (Solarbio, Beijing, China). Alkaline phosphatase (ALP) assays were conducted after 5-day osteogenic induction. ALP staining was carried out using a BCIP/NBT alkaline phosphatase color development kit (Beyotime, Shanghai, China). ALP activity was performed using alkaline phosphatase assay kit (Beyotime, Shanghai, China). Protein concentration of each sample was as described above. Alizarin red S (ARS) staining was performed after 2-week osteogenic induction using 0.2% ARS (Solarbio, Beijing, China) at 37 °C for 30 min. For semi-quantitative analysis, PBS containing 10% cetylpyridine chloride (Sigma-Aldrich, MO, USA) was added into each well to dissolve precipitation after staining and the absorbance was measured using a microplate reader (Tecan, Männedorf, Switzerland) at 542 nm. Cells were observed using an inverted microscope (Zeiss, Oberkochen, Germany).

### Detection of apoptosis

The percentage of apoptotic cells was examined after culture for 4 days using an Annexin V-FITC apoptosis detection kit (KeyGEN, Jiangsu, China). Briefly, cells were harvested, washed three times in cold PBS, resuspended in binding buffer at a concentration of 1 * 10^5^ cells/mL and incubated with 5 μL Annexin V-FITC and 5 μL PI for 15 min away from light at room temperature. Viable cells (Annexin V−/PI−), early apoptotic cells (Annexin V+/PI−), late apoptotic cells (Annexin V−/PI+ and necrotic cells (Annexin V+/PI+) were analyzed using flow cytometry (BD, NY, USA) and FlowJo (BD, NY, USA).

### Cell cycle and aneuploidy assay

With a cell cycle detection kit (KeyGEN, Jiangsu, China), cells that reached 70% confluence were used for the detection of cell cycle and cells that reached 90% confluence were used for the evaluation of aneuploidy. After trypsin digestion and centrifugation, precipitates were washed twice and resuspended with PBS into a single-cell suspension. Cold absolute alcohol was quickly added in the cell suspension and fixed cells at 4 °C for 24 h (final alcohol concentration of 70%). Finally, fixed cells were rinsed twice with PBS and stained with PI following instructions. Cell cycle and DNA aneuploidy were analyzed using flow cytometry (BD, NY, USA) and Modfit LT (VSH, USA).

### Cell senescence assay

The activity of senescence-associated β-galactosidase (SA-β-Gal) was evaluated using a cell senescence detection kit (Beyotime, Shanghai, China). hDFSCs (from passage 9 to passage 12) with or without NAC were cultured in 6-well plates and stained following the manufacturer’s protocol. Positive cells were observed in blue and calculated from 5 randomly selected fields using an inverted microscope (Zeiss, Oberkochen, Germany). Thereafter, the percentage of positive cells to whole cell number was calculated.

### Surface marker expression analysis

After culture for 4 days, hDFSCs were washed 3 times with PBS and collected using 0.25% trypsin with 1 mM EDTA at passage 6. Cell suspension (100,000 cells) was mixed with 5 μL of FITC-anti-CD44 (cat#338803, BioLegend, CA, USA) and PE-anti-CD90 (cat#328109, BioLegend, CA, USA) according to the manufacturer’s instructions. After incubation at room temperature for 30 min in the dark, the cells were washed 3 times with PBS and then acquired by flow cytometry (BD, NY, USA) and the MFI was analyzed by FlowJo (BD, NY, USA). For stem cell characterization, hDFSCs and rDFSCs at passage 3 were suspended in PBS. Cell suspension (100,000 cells) in 100 μL PBS was incubated with 5 μL of antibodies at room temperature for 30 min in the dark. Antibodies are listed in Additional file 6: Table S1. After washing twice with PBS, cells were analyzed via flow cytometry (BD, NY, USA) and FlowJo (BD, NY, USA).

### RNA extraction and qRT-PCR

After culture for 4 days, total RNA was extracted with TRIzol reagent (TransGen, Beijing, China) and a RNA kit (TransGen, Beijing, China). cDNA was synthesized with a First-Strand cDNA Synthesis Kit (TransGen, Beijing, China). The qPCR was carried on the 7500 Real Time PCR system (Thermo Fisher, MA, USA), using a green qPCR supermix (TranGen, Beijing, China). Results were analyzed and normalized to the housekeeping gene GAPDH using the ΔΔCt method. All primers (Sangon, Shanghai, China) are listed in Additional file 7: Table S2.

### Transcriptome analysis

After culture for 4 days, total RNA was extracted with TRIzol reagent (TransGen, Beijing, China), RNA sequencing was performed in collaboration with BGI-shenzhen following the standard operational procedure (https://www.genomics.cn/). The expression of human genes was performed by transforming mapped transcript reads to TPM. The significant levels were corrected by Q value with a threshold (*Q* value < 0.05) by Bonferroni. Differential expression analysis was performed using the DESeq2 (v1.4.5), Dr. Tom (BGI, Shenzhen, China) and R language (v4.0.3) ComplexHeatmap package (v2.4.3) with *Q* value < 0.05 and fold change > 1.5. Gene Ontology (GO) and Kyoto Encyclopedia of Genes and Genomes (KEGG) analysis were performed by Dr. Tom (BGI, Shenzhen, China) and R language TopGO package (v2.42.0) and ClusterProfiler package (v3.18.1).

### Western blot analysis

After culture for 4 days, cells were lysed in RIPA lysis buffer (Solarbio, Beijing, China) and 1 mM PMSF (Solarbio, Beijing, China) on ice. The concentration of protein was metered using the BCA Protein Assay Kit (Thermo Scientific, MA, USA) and heat denaturation of protein in protein sample loading buffer (EpiZyme, Shanghai, China) at 100 °C for 10 min. Equal quantities of protein were separated on 10% SDS-PAGE gels by electrophoresis, transferred onto 0.45 um polyvinylidene-fluoride membranes (PVDF) (Immobilon, MA, USA). Membranes were blocked with 5% (w/v) non-fat milk in TBST buffer pH 7.5 for 1 h at room temperature, incubated with the first antibody. Anti-GAPDH, PI3K p85, PI3K p110, AKT, phosphorylated-PI3K p85 and phosphorylated-AKT were used as primary antibodies. All antibodies are listed in Additional file 6: Table S1. Membranes were washed with TBST 3 times, 10 min each time, and incubated with corresponding secondary antibodies at room temperature for 1 h. Protein bands were visualized by ECL reagents (TransGen, Beijing, China).

### Rat tooth extraction model

The experimental protocol was approved by the Medical Ethics Committees of Stemmatological Hospital, Tianjin Medical University. The Unit for Laboratory Animal Medicine facilitated animal care at the Tianjin Hospital of ITCWM. This study conformed with the ARRIVE guidelines 2.0. Rat DFSCs (rDFSCs) were isolated from six unerupted mandible first molars from three 7-day-old SD rats according to our previous study [29] and characterization is shown in Additional file 3: Fig. S3. Antibodies are listed in Additional file 6: Table S1. NAC treatment was the same as before and cells at passage 6 were used for this experiment. Under anesthesia by intraperitoneal injection of 3% pentobarbital sodium (30 mg/kg), the right maxillary first molars were extracted on 20 Sprague–Dawley rats (male, 6-week-old) purchased from Beijing SPF Biotechnology. The sockets were formed using an 1# small ball drill to remove residue roots and bone pieces as much as possible, absorbable gelatin sponges (Xiang’en, Jiangxi, China) absorbed with PBS vehicle (15 μL), 5 mM NAC solution (15 μL), rDFSC suspension (10^6^ cells, 15 μL) or 5 mM NAC-treated rDFSC suspension (10^6^ cells, 15 μL) were placed into tooth extraction sockets depending on the randomized allocation of the animals, the sockets were finally sealed with tissue adhesive (*n*-butyl cyanoacrylate; 3 M, MN, USA) (Fig. 5A). A few animals with unsuccessful extraction were excluded. Only the experimenter who filled the sponges was aware of the group allocation. Based on different fillers, rats were divided into four groups (*n* = 4/group): CON (PBS vehicle), NAC (5 mM), CELL (rDFSCs), NAC + CELL (5-mM-NAC-treated rDFSCs). Four groups were always housed separately for the quality assurance of the treatment with the same housing condition and no diet modifications. Rats were humanely sacrificed by an overdose of anesthetics on postoperative day 7.

### Histologic assays

Right maxillary samples were fixed with 4% paraformaldehyde (Servicebio, Hubei, China) for 2 days at 4 °C, decalcified with EDTA solution (Servicebio, Hubei, China) for 21 days at room temperature, embedded in paraffin (Leica, Wetzlar, Germany) and cut into 4-μm-thick slices using a HistoCore AUTOCUT (Leica, Wetzlar, Germany). Extraction socket sections were stained with haematoxylin–eosin (HE) and masson trichrome following the manufacturer’s instructions (Servicebio, Hubei, China). Optical microscope (Nikon, Tokyo, Japan) was used to scan sections.

### Micro-computed tomography assessment

Right maxillary samples were fixed with 4% paraformaldehyde (Servicebio, Hubei, China) for 2 days at 4 °C, transferred to 70% alcohol, scanned using a SkyScan1276 system (BRUKER, MA, USA) at 10-μm scaled image pixel size with an energy level of 55 kV. The scans were reconstructed to produce three-dimensional images by CTvox (BRUKER, MA, USA) and two-dimensional section images by DataViewer (BRUKER, MA, USA). The extraction socket of the first molar was isolated by manual contouring and analyzed with regard to bone volume/tissue volume, bone mineral density and trabecular parameter (trabecular thickness, trabecular number, trabecular separation) by CTan software (BRUKER, MA, USA) at the consistent beginning grayscale index of 4.7.

### Statistical analysis

Data represented mean ± standard error (SEM) of independent samples. Statistical analysis was performed by Student’s t-test using GraphPad Prism 8 (GraphPad Software, CA, USA). Statistically significant differences between groups were determined by *p* < 0.05 (*), *p* < 0.01 (**), *p* < 0.001 (***), *p* < 0.0001 (****).

## Results

### Cytotoxic effects of NAC on hDFSCs

**Fig. 1:**
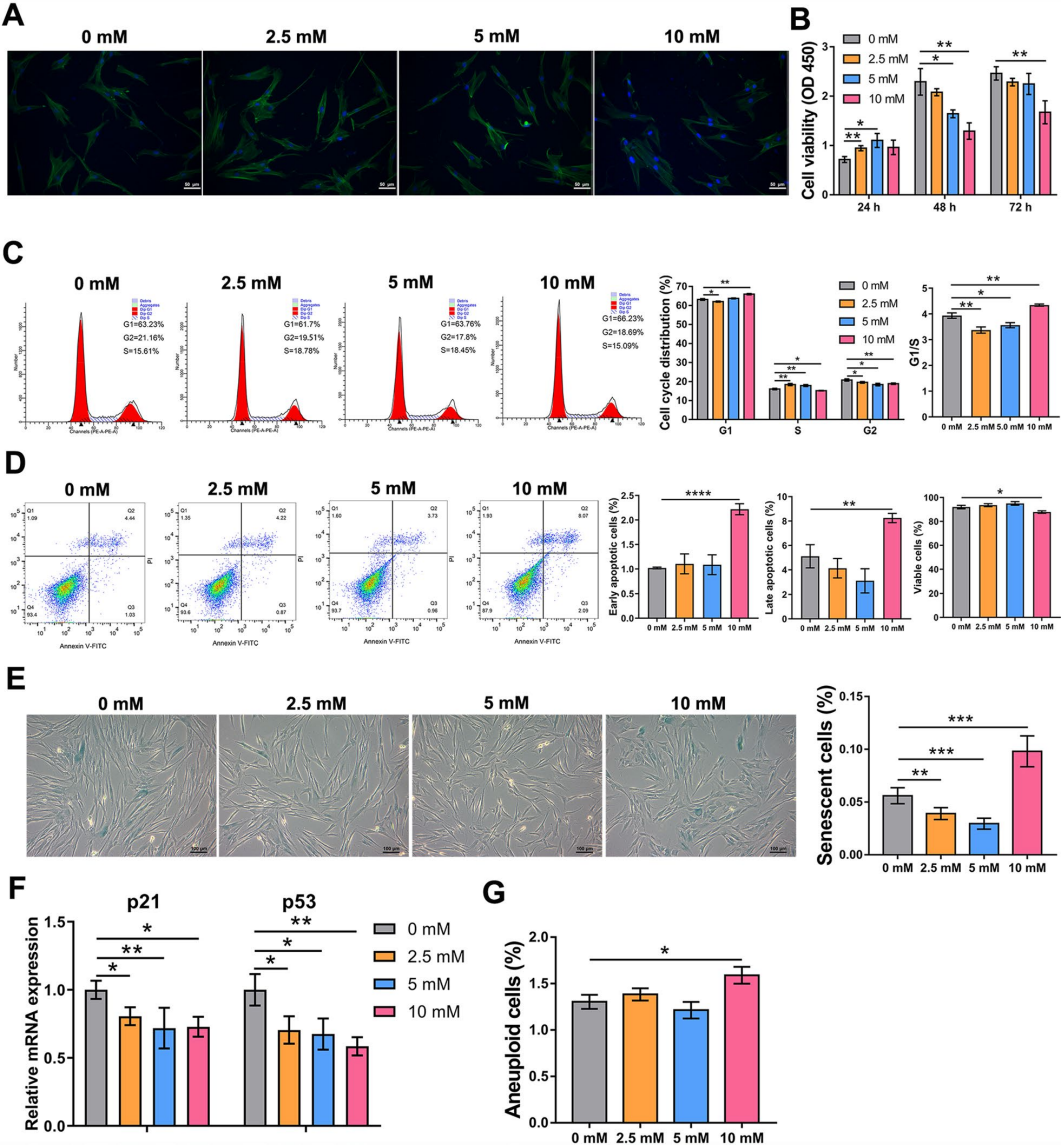
Cytotoxicity of NAC at various concentrations in hDFSCs. **A** Immunofluorescent staining of cytoskeleton structure (green for phalloidin, blue for DAPI) at day 3 after treatment. Scale bars: 50 μm. **B** Cell viability detected by CCK-8 assay at 24, 48 and 72 h. **C** Cell cycle picture and distribution detected by flow cytometry. **D** Apoptosis picture and the distribution of early and late apoptotic and viable cells detected by flow cytometry. **E** Photomicrographs and the distribution of blue-stained senescent cells of passage 12 using SA-β-Gal staining. Scale bars: 100 μm. **F** Relative mRNA expression of p21 and p53. **G** The distribution of aneuploid cells detected by flow cytometry. Statistically significant differences between groups were determined by *P* < 0.05 (*), *P* < 0.01 (**), *P* < 0.001 (***), *P* < 0.0001 (****)

hDFSCs stained with phalloidin exhibited a mesenchymal-like shape with a polygonal morphology which was not altered by NAC (Fig. 1A), cells treated with 2.5- or 5-mM NAC showed better viability than non-treated cells at 24 h and there was no distinct difference at 72 h, while addition of 10 mM NAC had no effect at 24 h but thereafter reduced viability (Fig. 1B). Flow cytometry showed decreased G1/S ratio and untriggered apoptosis in 2.5- or 5-mM NAC treatment; however, 10 mM NAC caused an increase in both G1/S ratio and apoptotic rate (Fig. 1C, D). Moreover, addition of 2.5- or 5-mM NAC decreased SA-β-gal-positive cell rate and 10 mM NAC increased the rate (Fig. 1E). Despite that relative mRNA levels of the tumor suppressor genes (p53, p21) decreased in all concentrations (Fig. 1F), only 10 mM NAC slightly increased the aneuploid cell rate (Fig. 1G).

### NAC enhances the properties of hDFSCs

**Fig. 2:**
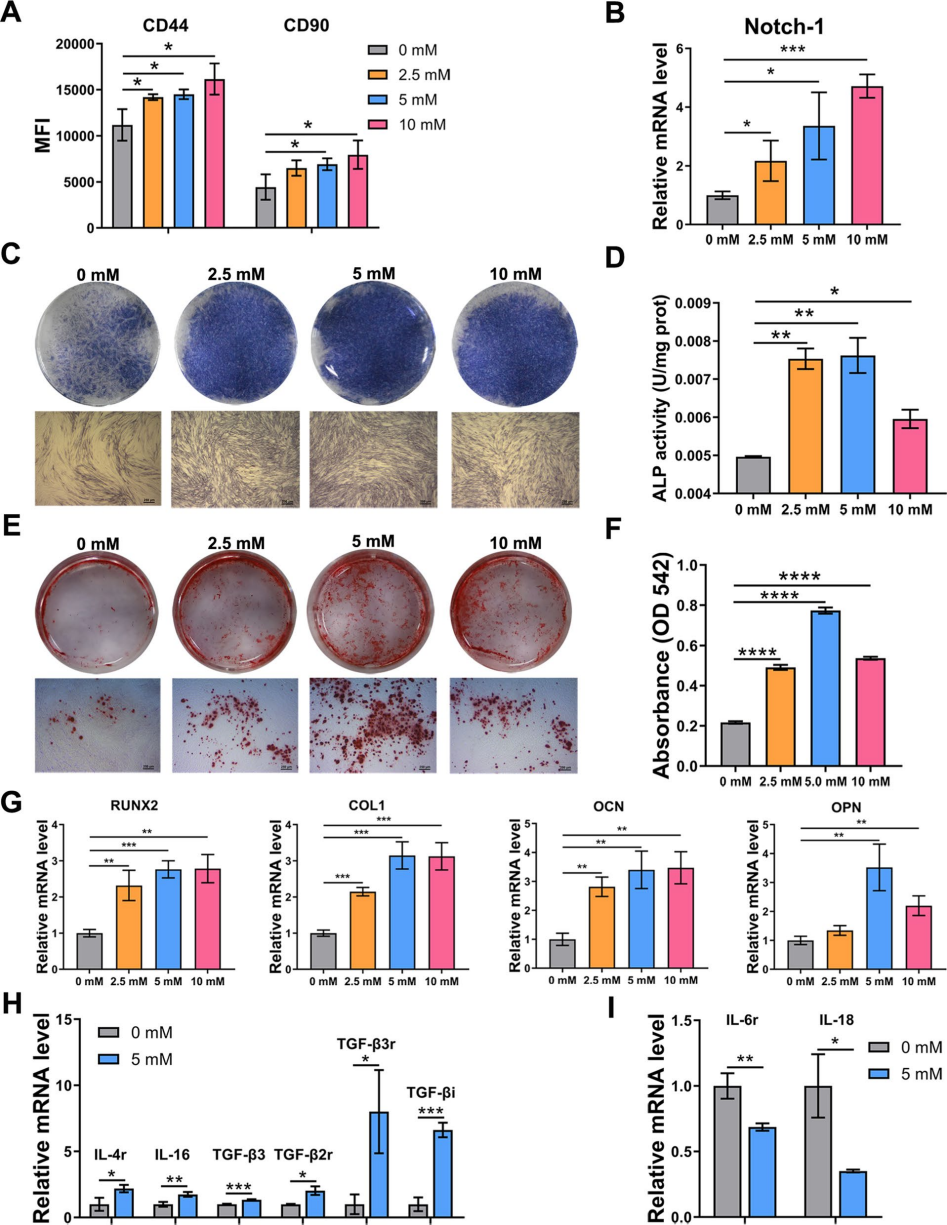
Effects of various NAC concentrations on stem cell-specific markers, osteogenesis and immune-related factors of hDFSCs. **A** MFI of CD44 and CD90 detected by flow cytometry. **B** Relative mRNA expression of Notch-1. **C** Photographs and micrographs depicting the osteogenic differentiation using ALP staining on day 7 after osteogenic induction. Scale bars: 250 μm. **D** Quantification of ALP activity. **E** Photographs and micrographs depicting the matrix mineralization using ARS staining on day 14 after osteogenic induction. Scale bars: 250 μm. **F** Semi-quantification of ARS staining. **G** Relative mRNA expression of osteogenic factors (RUNX2, COL1, OCN, OPN) after osteogenic culturing for 7 days. **H** Relative mRNA expression of anti-inflammation factors and receptors (IL-4r, IL-16, TGF-β3, TGF-β2r, TGF-β3r, TGF-βi). **I** Relative mRNA expression of pro-inflammation factors and receptors (IL-6r, IL-18). Statistically significant differences between groups were determined by *P* < 0.05 (*), *P* < 0.01 (**), *P* < 0.001 (***), *P* < 0.0001 (****)

The stem cell properties were assessed by stem cell-specific markers, osteogenic differentiation and immunomodulatory factors. The intensities of stem cell surface markers (CD44, CD90) (Fig. 2A) and the expression of hDFSC-specific factor (Notch-1) (Fig. 2B) were enhanced in NAC-treated hDFSCs. ALP (Fig. 2C, D) and ARS assays (Fig. 2E, F) showed that NAC increased osteogenic differentiation and calcium deposition; notably, the effect of 5 mM NAC was the best. The relative expression of osteogenic factors (RUNX2, COL1, OCN, OPN) was also upregulated after NAC treatment (Fig. 2G). Surprisingly, NAC treatment improved the stemness and osteogenesis of hDFSCs at passage 20 and the role of 10 mM in cells at passage 20 was more significant than its role in young cells (Additional file 4: Fig. S4). Furthermore, in 5 mM NAC-treated hDFSCs, anti-inflammatory IL-4 receptor (IL-4r), IL-16, TGF-β3, TGF-β2 receptor (TGF-β2r), TGF-β3 receptor (TGF-β3r) and TGF-β induced (TGF-βi) were fairly upregulated (Fig. 2H) while pro-inflammatory IL-6 receptor (IL-6r) and IL-18 were downregulated (Fig. 2I).

### Transcriptomic analysis and biological interpretation

**Fig. 3:**
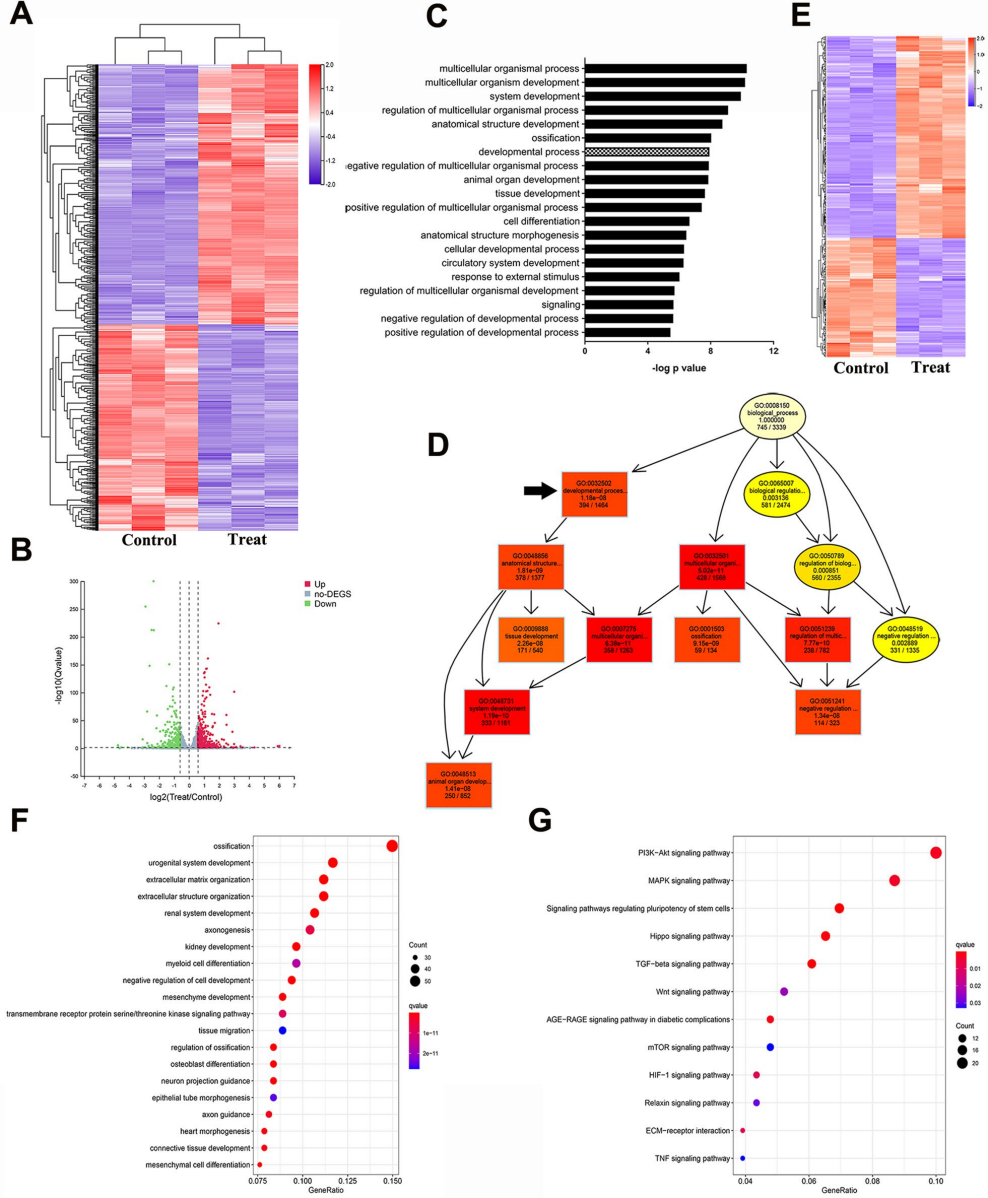
Differences in transcriptome signatures between controls and hDFSCs exposed to 5 mM NAC at day 4. DEGs visualized using a heatmap (**A**) and a volcano plot (**B**). **C** Graph depicting the first 20 GO terms of DEGs and their significance. **D** Graph depicting the regulatory relationship between the first 10 GO terms using DAG analysis. **E** Heatmap of 394 involved DEGs in “developmental process (GO: 0032502)”. **F** GO enrichment of DEGs in “developmental process (GO: 0032502)”. **G** KEGG enrichment of DEGs in “developmental process (GO: 0032502)”

Considering that 5 mM NAC-treated hDFSCs exhibited better performance in enhancing stem cell properties, we performed transcriptomic profiling between hDFSCs cultured with and without 5 mM NAC (SRA data: PRJNA780260) to understand the mechanistic insight into genetic alterations. 803 differentially expressed genes (DEGs) were observed in total, with 448 genes upregulated and 355 genes downregulated (Fig. 3A, B), and expression patterns of genes mentioned above were consistent with our transcriptomic data (Additional file 8: Table S3). 226 GO terms of biological process were significantly overrepresented (Additional file 9: Table S4) and the 20 most enriched GO terms were showed (Fig. 3C). The directed acyclic graph (DAG) further compared the membership of the first 10 GO terms with that of branches and pointed to the high hierarchical regulatory role of “developmental process (GO: 0,032,502)” (Fig. 3D).

### Enrichment analysis of “developmental process (GO: 0032502)”

Developmental potential is a key feature for the therapeutic purpose of a stem cell lineage [30–32], thus we screened 394 DEGs involved in “developmental process (GO: 0032502)” (Fig. 3E) and performed enrichment analysis based on Q value, gene number and rich ratio. GO enrichment analysis showed NAC could potentiate development of multiple tissues, especially bone tissue development (Fig. 3F). KEGG pathway analysis revealed a significant role of PI3K/AKT signal pathway in NAC-driven developmental events (Fig. 3G).

### NAC activates PI3K/AKT/ROS pathway

**Fig. 4:**
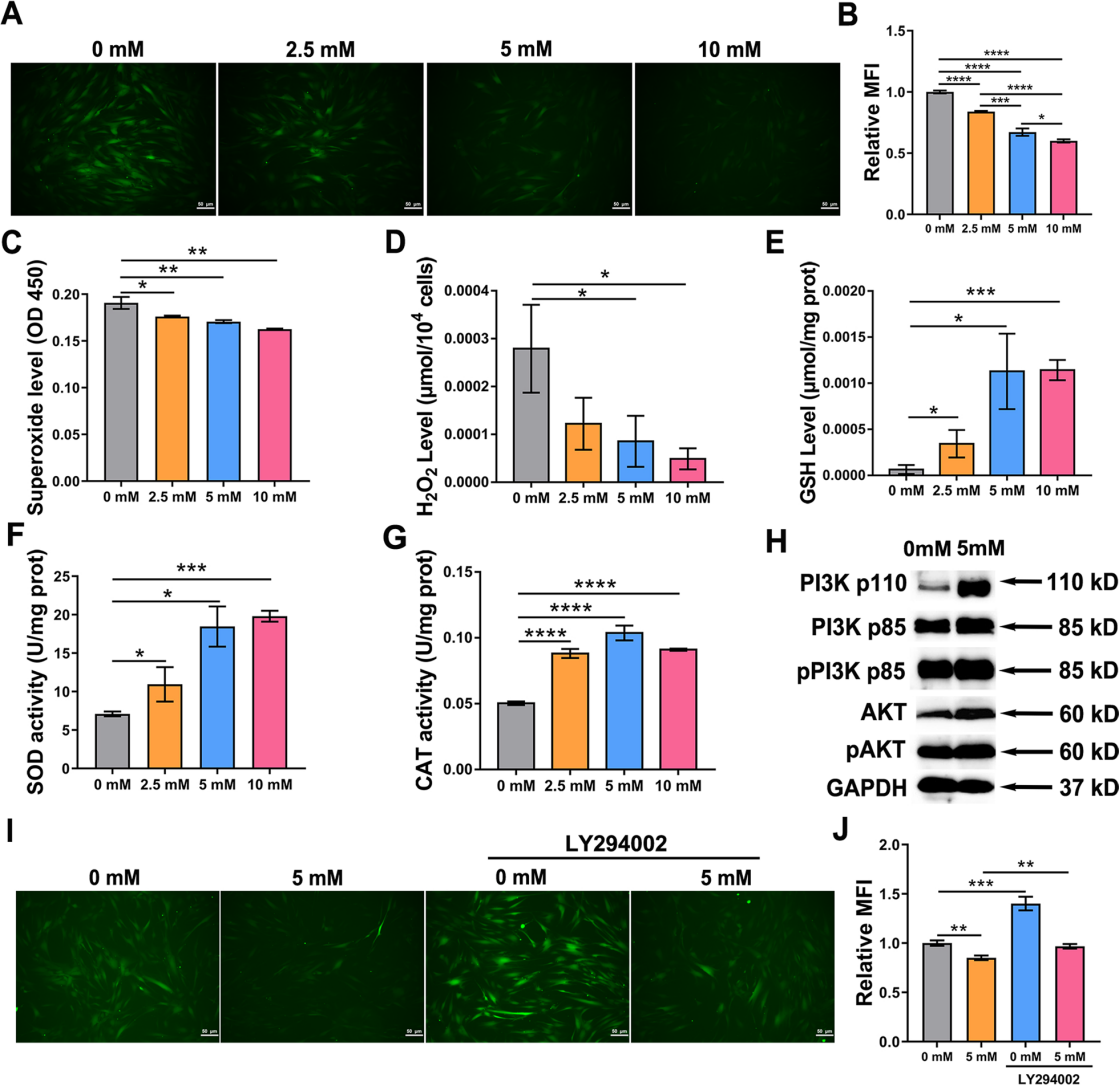
Effects of various NAC concentrations on ROS, antioxidant enzymes and PI3K/AKT pathway. **A** Fluorescent imaging depicting the intracellular ROS intensity. Scale bars: 50 μm. **B** Relative MFI of ROS intensity detected by flow cytometry. **C** Graph depicting the intracellular superoxide content. **D** Graph depicting the intracellular H_2_O_2_ content. **E** Graph depicting the intracellular GSH content. **F** Graph depicting the SOD activity. **G** Graph depicting the CAT activity. **H** Western blot depicting protein expression of PI3K-p85 (85 kD), PI3K-p110 (110 kD), AKT (60 kD), phosphorylated-PI3K-p85 (85 kD) and phosphorylated-AKT (60 kD) in hDFSCs treated with 5 mM NAC or not. **I** Fluorescent imaging depicting the intracellular ROS intensity after LY294002 treatment. Scale bars: 50 μm. **J** Relative MFI of ROS intensity after LY294002 treatment detected by flow cytometry. Statistically significant differences between groups were determined by *P* < 0.05 (*), *P* < 0.01 (**), *P* < 0.001 (***), *P* < 0.0001 (****)

To investigate underlying mechanisms, oxidant and antioxidant systems and PI3K/AKT signaling pathway were evaluated. After NAC incubation, ROS (Fig. 4A, B), superoxide (Fig. 4C) and H_2_O_2_ (Fig. 4D) were significantly decreased comparing with those in control passaged hDFSCs. Notably, the ROS level in 10 mM NAC-treated cells was the lowest (Fig. 4A, B). NAC replenished intracellular GSH contents (Fig. 4E) and increased the activities of two antioxidant enzymes, SOD (Fig. 4F) and CAT (Fig. 4G). Protein levels of PI3K-p85 (PI3K-regulatory subunit), PI3K-p110 (PI3K-catalytic subunit), AKT, phosphorylated-PI3K-p85 and phosphorylated-AKT increased in hDFSCs treated with 5 mM NAC (Fig. 4H; Additional file 5: Fig. S5A), consistent to KEGG enrichment (Fig. 3G) and TPM results (Additional file 8: Table S3). We then used LY294002, a PI3K functional inhibitor to suppress PI3K/AKT pathway and reevaluated ROS. LY294002 treatment caused the rebound of ROS in both control group and 5 mM NAC-treated group (Fig. 4I, J) due to the suppression of PI3K/AKT pathway (Additional file 5: Fig. S5B and C). ROS levels were decreased in NAC groups with and without LY294002 (Fig. 4I, J). These data indicate that NAC activates PI3K/AKT pathway to reinforce antioxidant defense and scavenge ROS.

### NAC facilitates alveolar bone repair in the tooth extraction socket

**Fig. 5:**
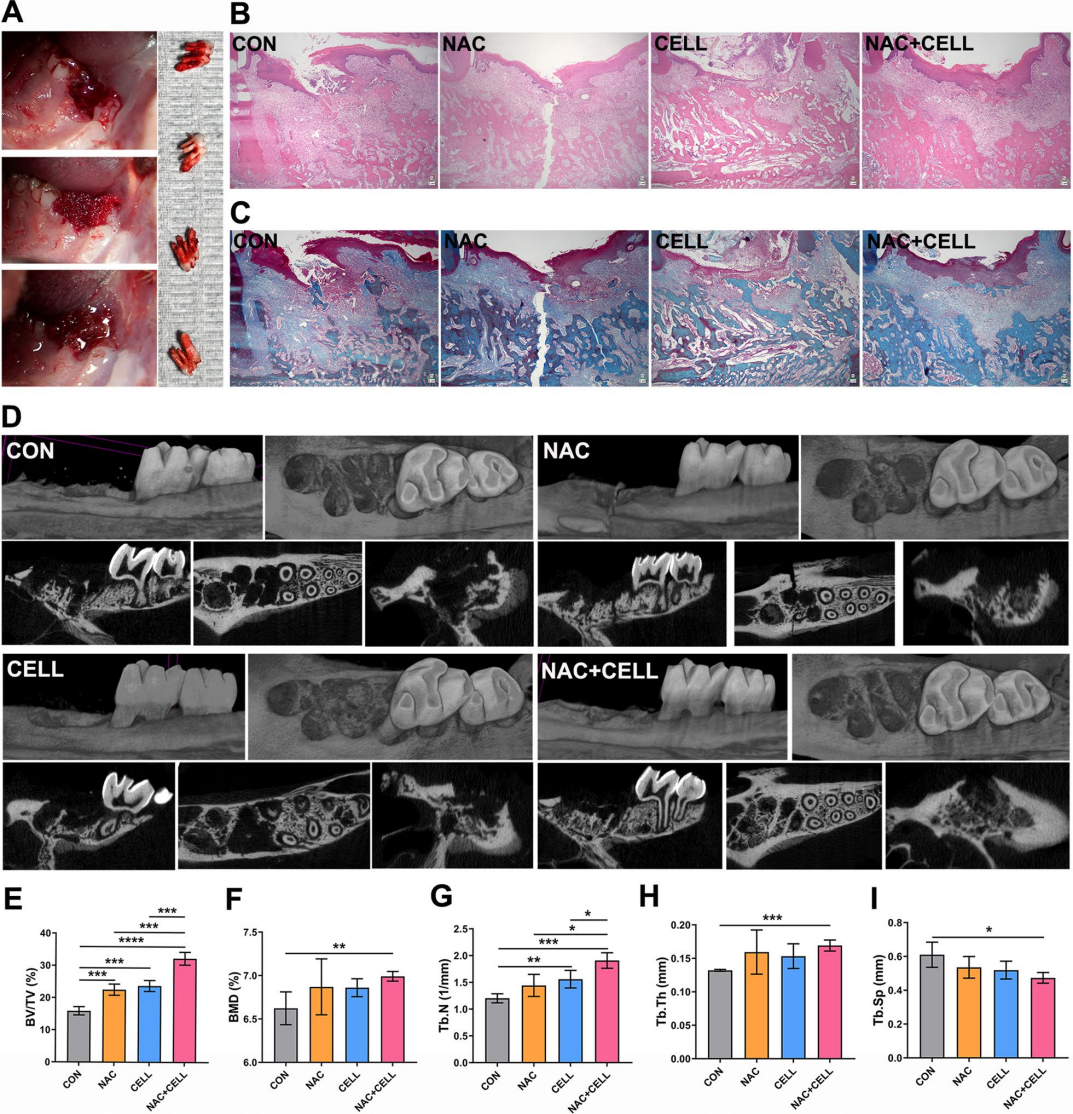
NAC-treated rDFSCs promoted tooth extraction socket bone formation. **A** The right maxillary first molars were extracted and filled with gelatin sponges carried vehicle (15 μL, PBS), NAC (15 μL, 5 mM), rDFSCs (15 μL, 1 × 10^6^ cells) or NAC-treated rDFSCs (15 μL, 5 mM NAC, 1 × 10^6^ cells), then sealed with cyanoacrylate glue. **B** Representative photomicrographs of HE-stained tooth extraction sockets. Original magnification: 4 × . **C** Representative photomicrographs of masson trichrome-stained tooth extraction sockets. Original magnification: 4 × . **D** Three-dimensional reconstruction and section images of tooth extraction sockets using micro-CT. Micro-CT assessment of BV/TV (**E**), BMD (**F**), Tb.Th (**G**), Tb.N (**H**), Tb.Sp (**I**). Statistically significant differences between groups were determined by *P* < 0.05 (*), *P* < 0.01 (**), *P* < 0.001 (***), *P* < 0.0001 (****)

**Fig. 6:**
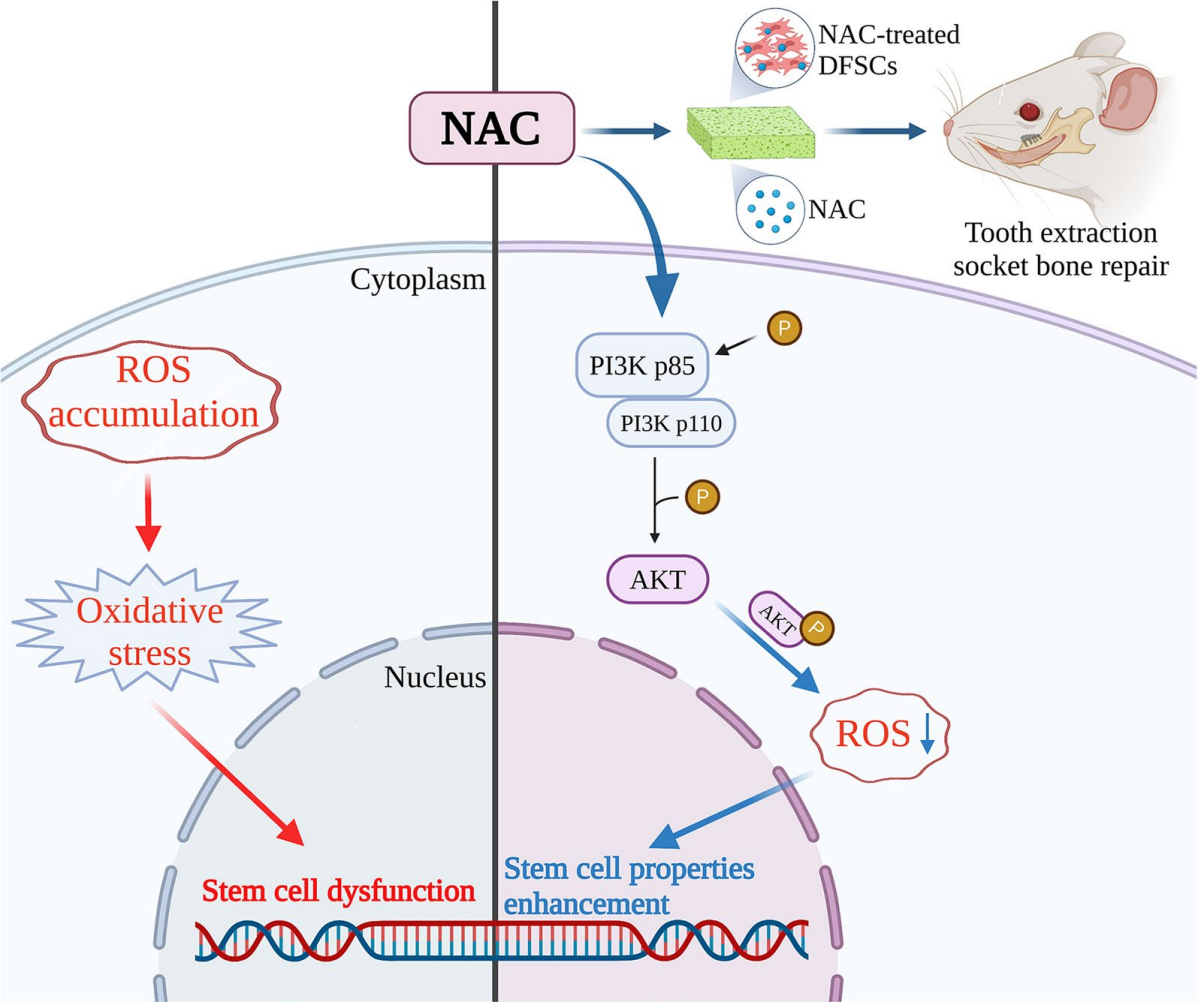
Schematic diagram of the proposed mechanism. NAC triggered PI3K/AKT signaling pathway that resulted in the reduction of ROS, leading to the enhancement of DFSC properties, NAC-treated DFSCs or NAC facilitated alveolar bone repair in the tooth extraction socket, created with BioRender.com

After extraction of the first molars, local transplantation of NAC, rDFSCs or NAC-treated rDFSCs exhibited acceleratory trends of wound closure (Additional file 10: Fig. S6), epithelium and bone repair without adverse signs (Fig. 5B). Masson trichrome staining showed similar trends (Fig. 5C). Three-dimensional reconstruction and sectional images also showed more osteoid formation in the sites filled with NAC, rDFSCs and NAC-treated rDFSCs than the control group (Fig. 5D). Quantitative assessment of first molar extraction sites confirmed that local transplantation of NAC, rDFSCs or NAC-treated rDFSCs promoted socket bone fill (15.89% vs 22.52% vs 23.72% vs 32.24%), with the highest potency of NAC-treated rDFSC transplantation (Fig. 5E). BMD increased only in the NAC-treated rDFSC group (6.623% vs 6.87% vs 6.86% vs 6.991%) (Fig. 5F). Additionally, NAC-treated rDFSC transplantation expressed a significant elevation in the trabecular thickness and number, together with a reduction in the trabecular separation, compared to the control group (Fig. 5G–I). Collectively, these data demonstrate that NAC triggers PI3K/AKT signaling pathway that resulted in the reduction of ROS, leading to the enhancement of DFSC properties, while NAC facilitates alveolar bone repair in the tooth extraction socket and achieves better in treatment with DFSCs (Fig. 6).

## Discussion

In this study, we have experimentally demonstrated that NAC regulates the properties of DFSCs via PI3K/AKT/ROS pathway. Additionally, we suggest a possibility that exogenous NAC is a putative contributing factor for alveolar bone repair in the field of dental stem cell-based therapy.

Studies often use H_2_O_2_ [20], high glucose [33] or hypoxia [34] to mimic oxidative stress and verify the antioxidative and cytoprotective effects of NAC. However, whether NAC would regulate stem cell properties by scavenging ROS accumulated during in vitro subculture merit further exploration. We observed that low concentrations of NAC (2.5- or 5-mM) promoted transient proliferation, but 10 mM NAC generated slight cytotoxicity which was consistent to previous studies [24, 35]. Our results further indicated that cell cycle arrest and apoptosis could be the cause of cytotoxicity. In addition, studies reported that antioxidant application could delay senescence and prevent stem cell dysfunction [13]. Indeed, we found that 2.5- or 5-mM NAC delayed senescence, but 10 mM NAC induced premature senescence. This result was similar to studies which illustrated premature senescence as one negative effect of high doses of antioxidants [35]. Oncogenic transformation is another concerned issue of stem cell-based therapy [36]. Downregulated p53 and p21 were observed after NAC treatment and 10 mM NAC increased aneuploid cell ratio, suggesting that DNA repair ability weakened with decreased ROS levels [37]. Notably, low concentrations of NAC did not affect gene stability. Taken together, re-evaluating the benefits of high dosage of antioxidants on stem cells seems important.

During tooth development, dental follicle develops into cementum and alveolar bone, so DFSCs possess strong directional osteogenic potential. In this study, expression of CD44, CD90 and Notch-1 as stemness factors typical of hDFSCs [38] increased with increasing NAC concentrations. Each NAC concentration promoted osteogenic differentiation in varying degrees, 5 mM showed the best osteogenic effect. Similarly, Masahiro Yamada also found 5 mM NAC was the most effective concentration for osteogenesis without apparent toxicity in bone marrow stem cells [21]. Additionally, altered expression levels of immune-related factors also indicated that 5 mM NAC contributed to the immunomodulatory properties of hDFSCs to some extent [39, 40]. The above results suggested that NAC improved the properties of hDFSCs during in vitro culture.

Transcriptome analysis was used to study the functional interaction and signal transduction networks of NAC in hDFSCs. Both GO term and DAG analysis revealed a significant association between NAC and developmental events. NAC has been reported as an osteogenesis-enhancing molecule for bone marrow stem cells [20, 21], our results confirm this point in hDFSCs and indicate broader regenerative potentials including extracellular matrix organization, urogenital and renal system development which need further investigation. We further explored underlying mechanisms of these applications. In hDFSCs, NAC not only served as a source of GSH but also activated primary antioxidant enzymes, SOD and CAT [41], leading to enhancement of antioxidant system and reduction of ROS. KEGG enrichment shed light on the PI3K/AKT pathway as a key mechanism for NAC-mediated effects on hDFSCs. The PI3K/AKT signaling is a critical ROS-dependent pathway and strongly links with stem cell properties [42, 43]. Upregulations of PI3K, AKT and their phosphorylation forms after NAC treatment were confirmed by western blot. More importantly, ROS rebounded in both groups and NAC still exhibited the antioxidative effect following PI3K inhibition, suggesting that PI3K/AKT signaling contributed to the antioxidative mechanism of NAC and there were other, as yet unknown molecular mechanisms that remained to be examined. PI3K/AKT pathway was thought to be mediated by ROS [43], paradoxically we found the activation of PI3K/AKT pathway could inhibit ROS in turn. We speculated that there was a feedback loop between ROS and PI3K/AKT pathway in regulating stem cell properties. This needs to be further researched.

Most previous literature reports that various NAC concentrations preserve stem cell function by inhibiting oxidative stress induced by ROS. In current study, the effects of NAC on hDFSC properties are not always in positive correlation with NAC concentrations. High concentrations (such as 10 mM) showed suboptimal osteogenic ability even with mild cytotoxicity, as mentioned in a few studies [21, 24]. The presence of reductive stress may be the reason. Reduced ROS levels are critical for maintaining stem cell identity, meanwhile a persistent lack of ROS, or reductive stress, also impedes stem cell functioning [44–46]. This may explain why 10 mM NAC minimizes the intracellular ROS level, but 5 mM NAC shows better results. These findings imply that cellular antioxidant defenses are saturated when an antioxidant reaches a certain concentration, after exceeding this concentration stem cells generate reductive stress from overdosed antioxidants.

Finally, we use rodent tooth extraction model to investigate the pro-osteogenic effect of NAC by itself or with rDFSCs. Ideal tooth extraction socket bone repair is necessary for any prosthodontic therapies. However, the oral cavity is exposed to ROS produced by many oxidizing agents, such as oral microflora and mechanical injuries [47–49], leading to delayed and undesirable bone healing in the sites after tooth extraction. In this study, exogenous supplementation of rDFSCs or NAC at an optimal concentration promoted tooth extraction socket bone formation and NAC-treated rDFSCs were superior. Therefore, NAC may be of therapeutic benefit in alveolar bone regeneration. To simulate the clinical situation, the current study uses topical administration carried by the gelatin sponge instead of systemic injection [50]. The gelatin sponge carrier and the tissue glue are designed to deliver drugs deep into the extraction socket, seal the wound and assist in blood clot formation, which may have a limited healing effect. Clearly, further studies are needed to explore the pharmacology of NAC and stem cells carried by gelatin sponge.

## Conclusions

In summary, this study showed that the proper concentration of NAC enhances the properties of DFSCs, especially osteogenesis, via PI3K/AKT/ROS signaling, and promotes alveolar bone repair by itself or with DFSCs. We also provide evidence for the effect of the reductive stress in stem cells caused by excessive antioxidants. NAC as a safe and FDA-approved drug has multiple advantages in clinical applications of dentistry. Thus, it will be of great interest to study the role, mechanism and appropriate usage of NAC in dental stem cell biology and alveolar bone regenerative medicine.

## Supplementary Information

**Additional file 1. Fig. S1: **The graphical overview of NAC and dental follicles. (A) Graph depicting the molecular formula and the direct and indirect antioxidant effects of NAC, created with BioRender.com. (B) Graph depicting the dental follicles wrapped unerupted third molars, created with BioRender.com. (C) The clinical imaging and radiographic imaging of dental follicles.

**Additional file 2. Fig. S2: **Characterization and morphology of human dental follicle stem cells (hDFSCs). (A) The strategy of gating for flow cytometry analysis. (B) Negative markers including CD31, CD117. (C) Positive markers including CD29, CD44, CD90. (D) Representative images of immunofluorescence staining of hDFSCs with the mesenchymal marker (positive for Vimentin; green) and nuclei (DAPI; blue). Scale bars: 50 μm. (E) Representative images of immunofluorescence staining of hDFSCs with the epithelial marker (negative for CK14; green) and nuclei (DAPI; blue). Scale bars: 50 μm. (F) Osteogenic differentiation. Representative images of alkaline phosphatase staining after osteogenic culturing for 5 days. Scale bars: 250 μm. (G) Matrix mineralization. Representative images of alizarin red s staining after osteogenic culturing for 15 days. Scale bars: 250 μm. (H) Adipogenic differentiation. Representative images of oil red o staining after adipogenic induction for 15 days. Scale bars: 100 μm. (I) Neurogenic differentiation potential. Representative images of immunofluorescence staining with neurogenic marker (positive for β-III-tubulin; red) and nuclei (DAPI; blue). Scale bars: 50 μm. (J) The cellular density and morphology of hDFSCs treated with different concentrations of NAC and control cells at day 1, day 2 and day 3 under the light microscope. Scale bars: 250 μm and 50 μm.

**Additional file 3. Fig. S3: **Characterization of rat dental follicle stem cells (rDFSCs). (A) The strategy of gating for flow cytometry analysis. (B) Positive markers including CD29, CD90. (C) Negative markers including CD11, CD45, CD106. (D) Representative images of immunofluorescence staining of rDFSCs with the mesenchymal marker (positive for Vimentin; green) and nuclei (DAPI; blue). Scale bars: 100 μm. (E) Representative images of immunofluorescence staining of rDFSCs with the epithelial marker (negative for CK14; green) and nuclei (DAPI; blue). Scale bars: 100 μm. (F) Osteogenic differentiation. Representative images of alkaline phosphatase staining after osteogenic culturing for 5 days. Scale bars: 250 μm. (G) Matrix mineralization. Representative images of alizarin red s staining after osteogenic culturing for 15 days. Scale bars: 100 μm. (H) Adipogenic differentiation. Representative images of oil red o staining after adipogenic induction for 15 days. Scale bars: 50 μm. (I) Neurogenic differentiation potential. Representative images of immunofluorescence staining with neurogenic marker (positive for β-III-tubulin; red) and nuclei (DAPI; blue). Scale bars: 50 μm.

**Additional file 4. Fig. S4: **Effects of various NAC concentrations on stem cell-specific markers and osteogenesis of hDFSCs at passage 20 (non-treated cells at passage 9 and 20 were used as the control). (A) MFI of CD44 and CD90 detected by flow cytometry. (B) Relative mRNA expression of Notch-1. (C) Photographs and micrographs depicting the osteogenic differentiation using ALP staining on day 5 after osteogenic induction. Scale bars: 250 μm. (D) Quantification of ALP activity. (E) Photographs and micrographs depicting the matrix mineralization using ARS staining on day 15 after osteogenic induction. Scale bars: 250 μm. (F) Semi-quantification of ARS staining. (G) Relative mRNA expression of osteogenic factors (RUNX2, COL1, OCN, OPN) after osteogenic culturing for 7 days. Statistically significant differences between groups were determined by P < 0.05 (*), P < 0.01 (**), P < 0.001 (***), P < 0.0001 (****).

**Additional file 5. Fig. S5: **Grayscale graph analysis of PI3K/AKT pathway proteins. (A) Grayscale graph analysis of PI3K/AKT pathway proteins after NAC treatment. (B) Representative western blots of PI3K/AKT pathway proteins after LY294002 treatment. (C) Grayscale graph analysis of PI3K/AKT pathway proteins after LY294002 treatment.

**Additional file 6. Table S1: **Antibodies used in this study.

**Additional file 7. Table S2: **Primer sequences used in this study.

**Additional file 8. Table S3: **TPM of genes discussed in the text.

**Additional file 9. Table S4: **GO enriched terms of biological process.

**Additional file 10. Fig. S6: **Tooth extraction socket wounds. (A) Photographs of tooth extraction socket wounds at day 7 post-extraction. (B) Wound areas analyzed by Image J. Statistically significant differences between groups were determined by P < 0.05 (*), P < 0.01 (**), P < 0.001 (***), P < 0.0001 (****).

## Data Availability

The datasets used and/or analyzed during the current study are available from the corresponding author on reasonable request. SRA records of RNA-sequencing will be accessible with the following link after 2022-12-01: https://www.ncbi.nlm.nih.gov/sra/PRJNA780260.

## Abbreviations

DFSCs: Dental follicle stem cells
hDFSCs: Human dental follicle stem cells
rDFSCs: Rat dental follicle stem cells
NAC: *N*-acetylcysteine
ROS: Reactive oxygen species
GSH: Glutathione
RNA: Ribonucleic acid
HEPES: *N*-2-hydroxyethylpiperazine-*N*-ethane-sulphonicacid
α-MEM: Minimum essential medium-α
FBS: Fetal bovine serum
PBS: Phosphate buffer solution
EDTA: Ethylene diamine tetraacetic acid
PI3K: Phosphatidylinositol 3 kinase
DCFH-DA: 2′,7′-Dichlorofluorescent yellow diacetate
MFI: Mean fluorescence intensity
H_2_O_2_: Hydrogen peroxide
SOD: Superoxide dismutase
CAT: Catalase
CCK-8: Cell counting kit-8
ALP: Alkaline phosphatase
ARS: Alizarin red S
SA-β-Gal: Senescence-associated β-galactosidase
FITC: Fluorescein isothiocyanate
PI: Propidium iodide
PE: Phycoerythrin
TPM: Transcripts per million
AKT: Protein kinase B
HE: Haematoxylin and eosin
IL: Interleukin
TGF-β: Transforming growth factor-β
DEGs: Differentially expressed genes
GO: Gene ontology
DAG: Directed acyclic graph
KEGG: Kyoto encyclopedia of genes and genomes
Micro-CT: Micro-computed tomography
BV/TV: Bone volume/tissue volume
BMD: Bone mineral density
Tb.Th: Trabecular thickness
Tb.N: Trabecular number
Tb.Sp: Trabecular separation
