# Supplementary figures and images for: N-acetylcysteine regulates dental follicle stem cell osteogenesis and alveolar bone repair via ROS scavenging
Source: Stem Cell Res Ther. 2022 Sep 8;13:466. doi: 10.1186/s13287-022-03161-y (PMC9461171; doi:10.1186/s13287-022-03161-y)

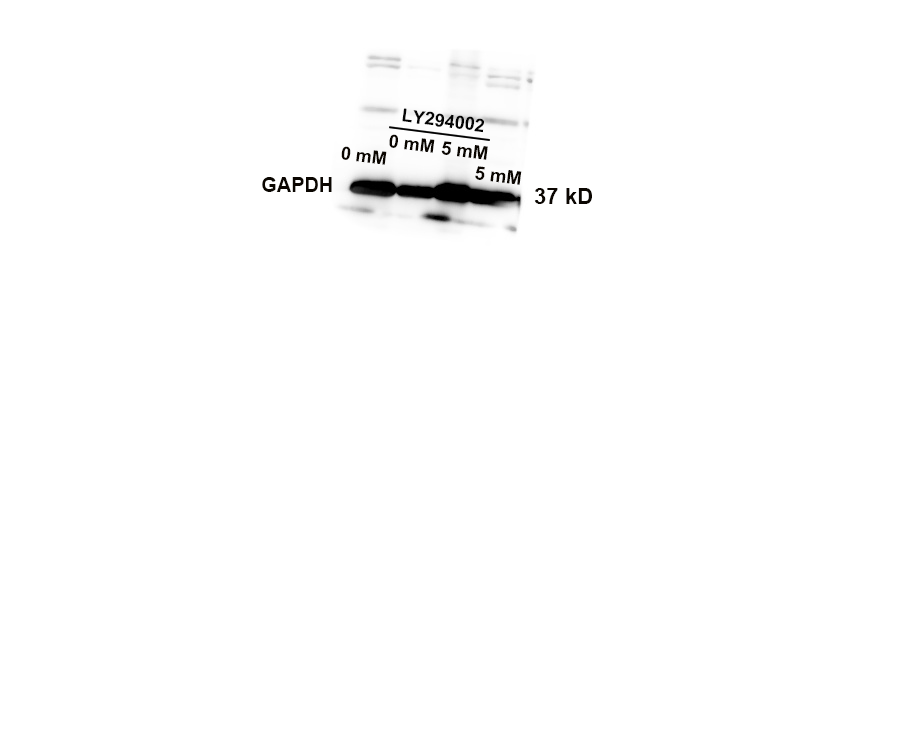

Supplement: Supplementary file 1 — Additional file 1. Fig. S1: The graphical overview of NAC and dental follicles. (A) Graph depicting the molecular formula and the direct and indirect antioxidant effects of NAC, created with BioRender.com. (B) Graph depicting the dental follicles wrapped unerupted third molars, created with BioRender.com. (C) The clinical imaging and radiographic imaging of dental follicles. [file 13287_2022_3161_MOESM1_ESM.doc]

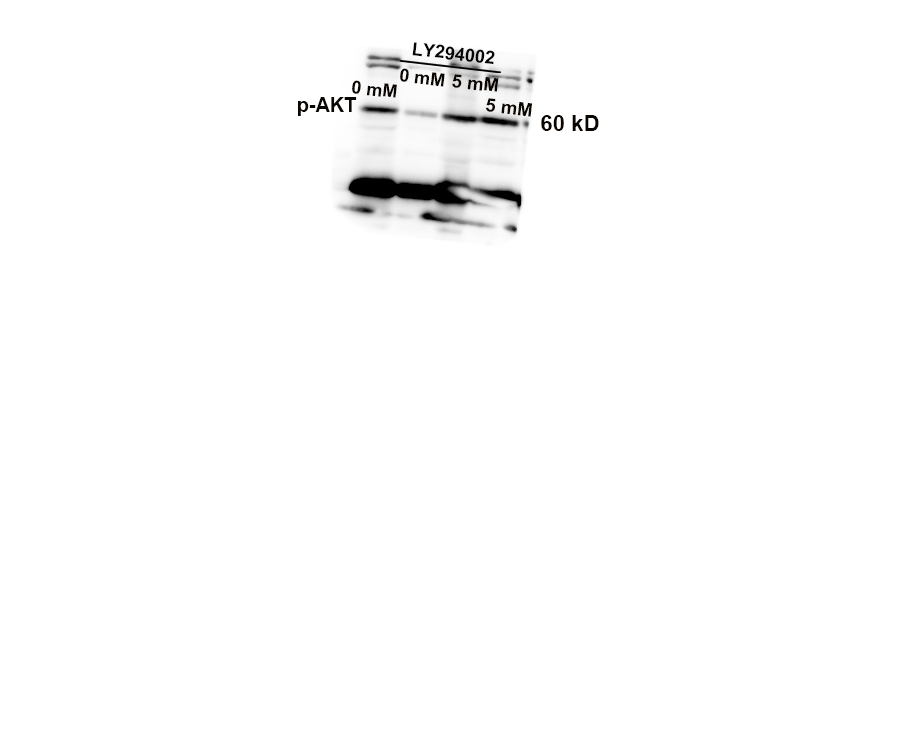

Supplement: Supplementary file 2 — Additional file 2. Fig. S2: Characterization and morphology of human dental follicle stem cells (hDFSCs). (A) The strategy of gating for flow cytometry analysis. (B) Negative markers including CD31, CD117. (C) Positive markers including CD29, CD44, CD90. (D) Representative images of immunofluorescence staining of hDFSCs with the mesenchymal marker (positive for Vimentin; green) and nuclei (DAPI; blue). Scale bars: 50 μm. (E) Representative images of immunofluorescence staining of hDFSCs with the epithelial marker (negative for CK14; green) and nuclei (DAPI; blue). Scale bars: 50 μm. (F) Osteogenic differentiation. Representative images of alkaline phosphatase staining after osteogenic culturing for 5 days. Scale bars: 250 μm. (G) Matrix mineralization. Representative images of alizarin red s staining after osteogenic culturing for 15 days. Scale bars: 250 μm. (H) Adipogenic differentiation. Representative images of oil red o staining after adipogenic induction for 15 days. Scale bars: 100 μm. (I) Neurogenic differentiation potential. Representative images of immunofluorescence staining with neurogenic marker (positive for β-III-tubulin; red) and nuclei (DAPI; blue). Scale bars: 50 μm. (J) The cellular density and morphology of hDFSCs treated with different concentrations of NAC and control cells at day 1, day 2 and day 3 under the light microscope. Scale bars: 250 μm and 50 μm. [file 13287_2022_3161_MOESM2_ESM.doc]

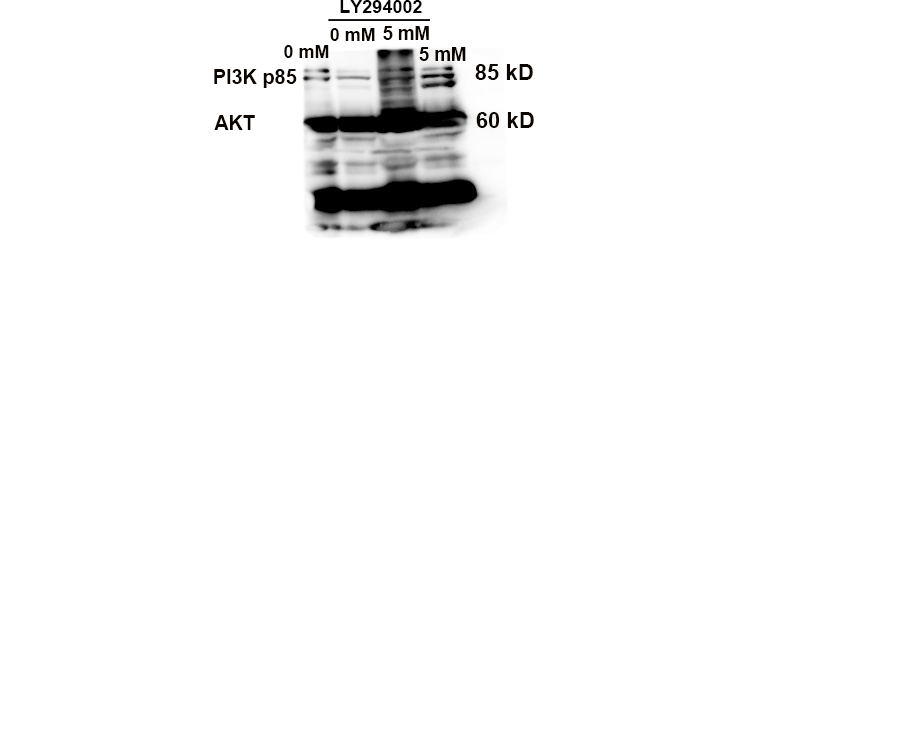

Supplement: Supplementary file 3 — Additional file 3. Fig. S3: Characterization of rat dental follicle stem cells (rDFSCs). (A) The strategy of gating for flow cytometry analysis. (B) Positive markers including CD29, CD90. (C) Negative markers including CD11, CD45, CD106. (D) Representative images of immunofluorescence staining of rDFSCs with the mesenchymal marker (positive for Vimentin; green) and nuclei (DAPI; blue). Scale bars: 100 μm. (E) Representative images of immunofluorescence staining of rDFSCs with the epithelial marker (negative for CK14; green) and nuclei (DAPI; blue). Scale bars: 100 μm. (F) Osteogenic differentiation. Representative images of alkaline phosphatase staining after osteogenic culturing for 5 days. Scale bars: 250 μm. (G) Matrix mineralization. Representative images of alizarin red s staining after osteogenic culturing for 15 days. Scale bars: 100 μm. (H) Adipogenic differentiation. Representative images of oil red o staining after adipogenic induction for 15 days. Scale bars: 50 μm. (I) Neurogenic differentiation potential. Representative images of immunofluorescence staining with neurogenic marker (positive for β-III-tubulin; red) and nuclei (DAPI; blue). Scale bars: 50 μm. [file 13287_2022_3161_MOESM3_ESM.doc]

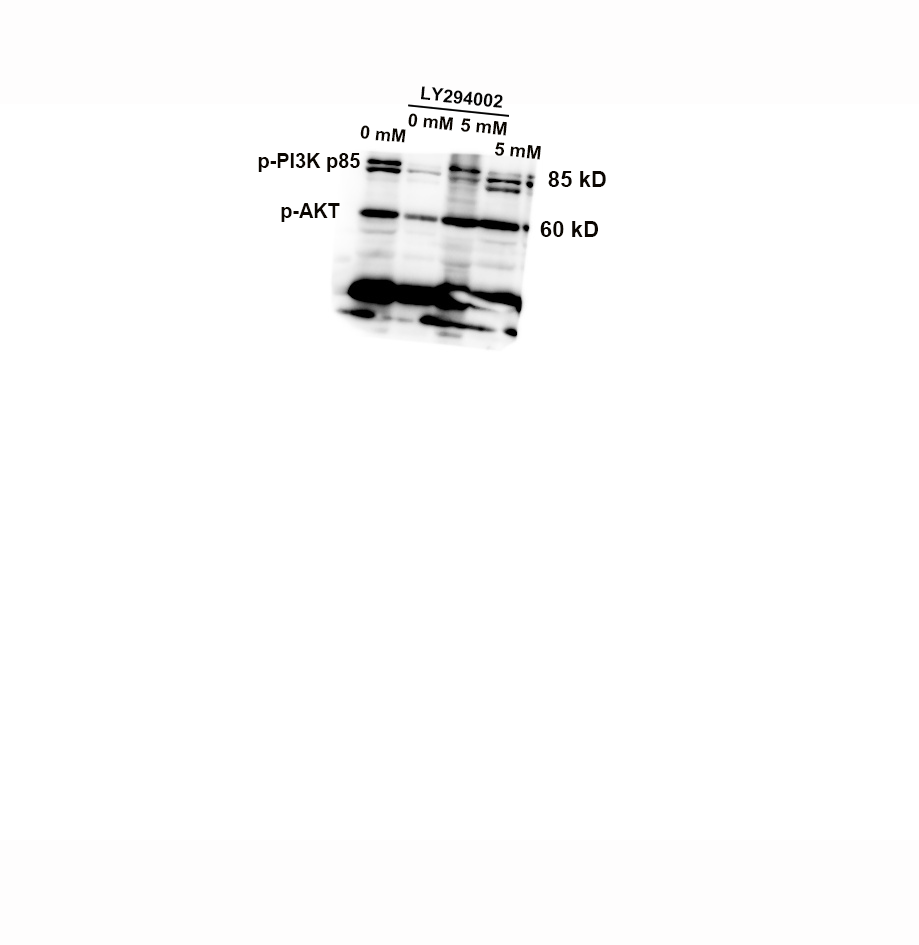

Supplement: Supplementary file 4 — Additional file 4. Fig. S4: Effects of various NAC concentrations on stem cell-specific markers and osteogenesis of hDFSCs at passage 20 (non-treated cells at passage 9 and 20 were used as the control). (A) MFI of CD44 and CD90 detected by flow cytometry. (B) Relative mRNA expression of Notch-1. (C) Photographs and micrographs depicting the osteogenic differentiation using ALP staining on day 5 after osteogenic induction. Scale bars: 250 μm. (D) Quantification of ALP activity. (E) Photographs and micrographs depicting the matrix mineralization using ARS staining on day 15 after osteogenic induction. Scale bars: 250 μm. (F) Semi-quantification of ARS staining. (G) Relative mRNA expression of osteogenic factors (RUNX2, COL1, OCN, OPN) after osteogenic culturing for 7 days. Statistically significant differences between groups were determined by P < 0.05 (*), P < 0.01 (**), P < 0.001 (***), P < 0.0001 (****). [file 13287_2022_3161_MOESM4_ESM.doc]
