# Supplementary material for: N-acetylcysteine regulates dental follicle stem cell osteogenesis and alveolar bone repair via ROS scavenging
Source: Stem Cell Res Ther. 2022 Sep 8;13:466. doi: 10.1186/s13287-022-03161-y (PMC9461171; doi:10.1186/s13287-022-03161-y)
Supplement: Supplementary file 5 — Additional file 5. Fig. S5: Grayscale graph analysis of PI3K/AKT pathway proteins. (A) Grayscale graph analysis of PI3K/AKT pathway proteins after NAC treatment. (B) Representative western blots of PI3K/AKT pathway proteins after LY294002 treatment. (C) Grayscale graph analysis of PI3K/AKT pathway proteins after LY294002 treatment. [file 13287_2022_3161_MOESM5_ESM.doc]

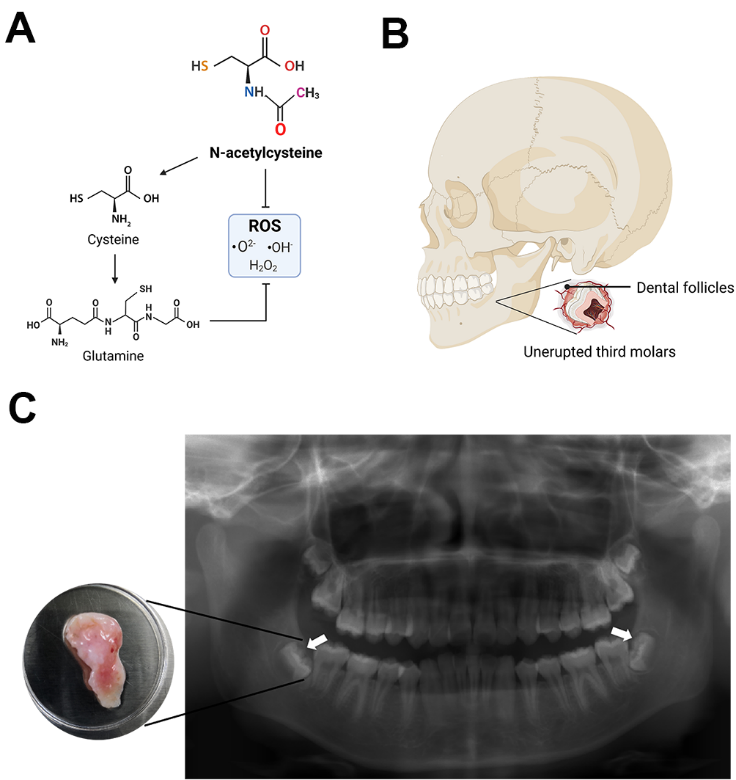


Supplementary Figure 1. The graphical overview of NAC and human dental follicles. **(A)** Graph depicting the molecular formula and the direct and indirect antioxidant effects of NAC, created with BioRender.com. **(B)** Graph depicting the human dental follicles wrapped unerupted third molars, created with BioRender.com. **(C)** The clinical imaging and radiographic imaging of human dental follicles.
