# Supplementary material for: N-acetylcysteine regulates dental follicle stem cell osteogenesis and alveolar bone repair via ROS scavenging
Source: Stem Cell Res Ther. 2022 Sep 8;13:466. doi: 10.1186/s13287-022-03161-y (PMC9461171; doi:10.1186/s13287-022-03161-y)
Supplement: Supplementary file 7 — Additional file 7. Table S2: Primer sequences used in this study. [file 13287_2022_3161_MOESM7_ESM.doc]

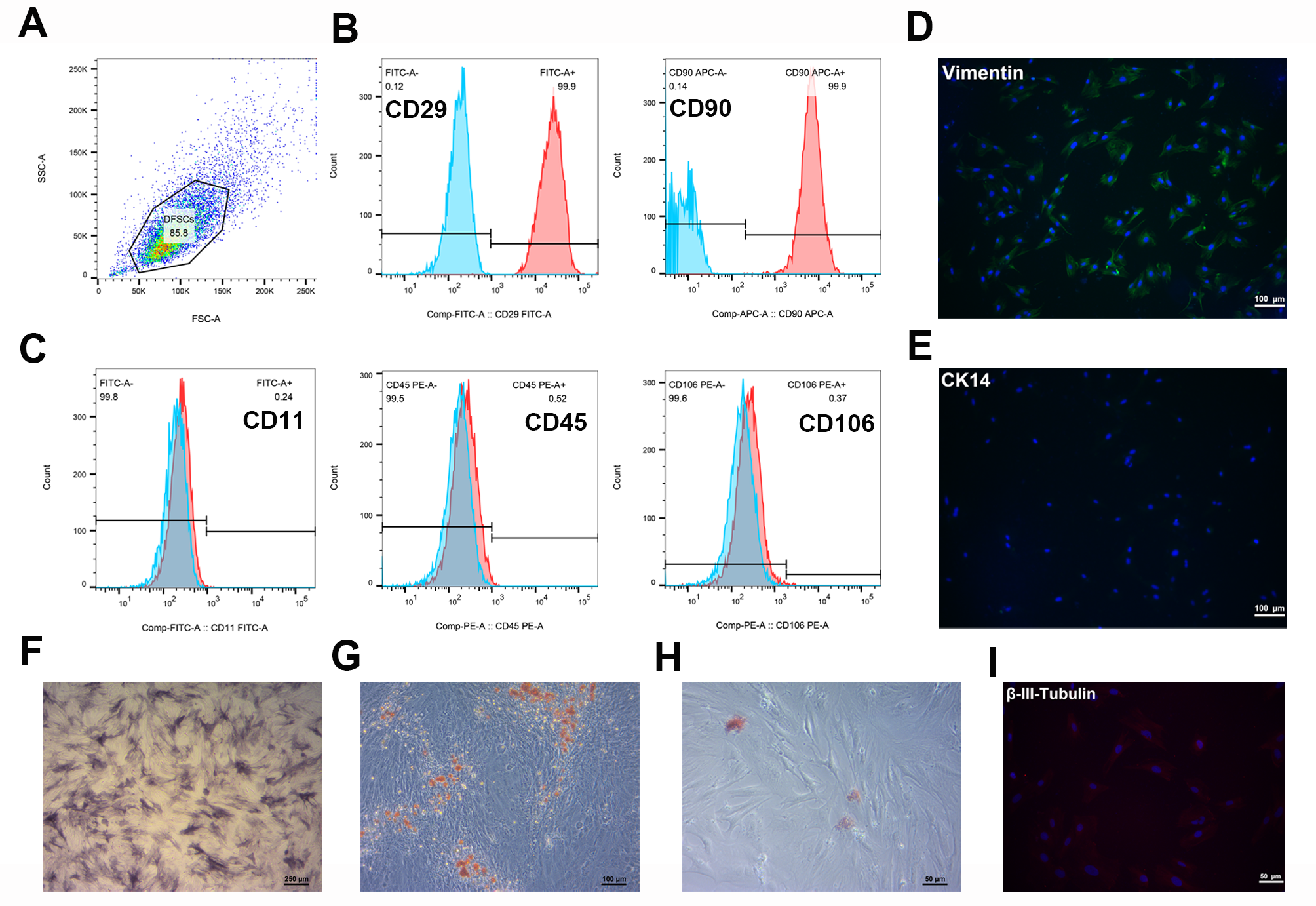


Supplementary Figure 3. Characterization of rat dental follicle stem cells (rDFSCs). **(A)** The strategy of gating for flow cytometry analysis. **(B)** Positive markers involve CD29, CD90. **(C)** Negative markers including CD11, CD45, CD106. **(D)** Representative images of immunofluorescence staining of rDFSCs with the mesenchymal marker (positive for Vimentin; green) and nuclei (DAPI; blue). Scale bars: 100 μm. **(E)** Representative images of immunofluorescence staining of rDFSCs with the epithelial marker (negative for CK14; green) and nuclei (DAPI; blue). Scale bars: 100 μm. **(F)** Osteogenic differentiation. Representative images of alkaline phosphatase staining after osteogenic culturing for 5 days. Scale bars: 250 μm. **(G)** Matrix mineralization. Representative images of alizarin red s staining after osteogenic culturing for 15 days. Scale bars: 100 μm. **(H)** Adipogenic differentiation. Representative images of oil red o staining after adipogenic induction for 15 days. Scale bars: 50 μm. **(I)** Neurogenic differentiation potential. Representative images of immunofluorescence staining with neurogenic marker (positive for β-III-tubulin; red) and nuclei (DAPI; blue). Scale bars: 50 μm.
