# Supplementary material for: N-acetylcysteine regulates dental follicle stem cell osteogenesis and alveolar bone repair via ROS scavenging
Source: Stem Cell Res Ther. 2022 Sep 8;13:466. doi: 10.1186/s13287-022-03161-y (PMC9461171; doi:10.1186/s13287-022-03161-y)
Supplement: Supplementary file 8 — Additional file 8. Table S3: TPM of genes discussed in the text. [file 13287_2022_3161_MOESM8_ESM.doc]

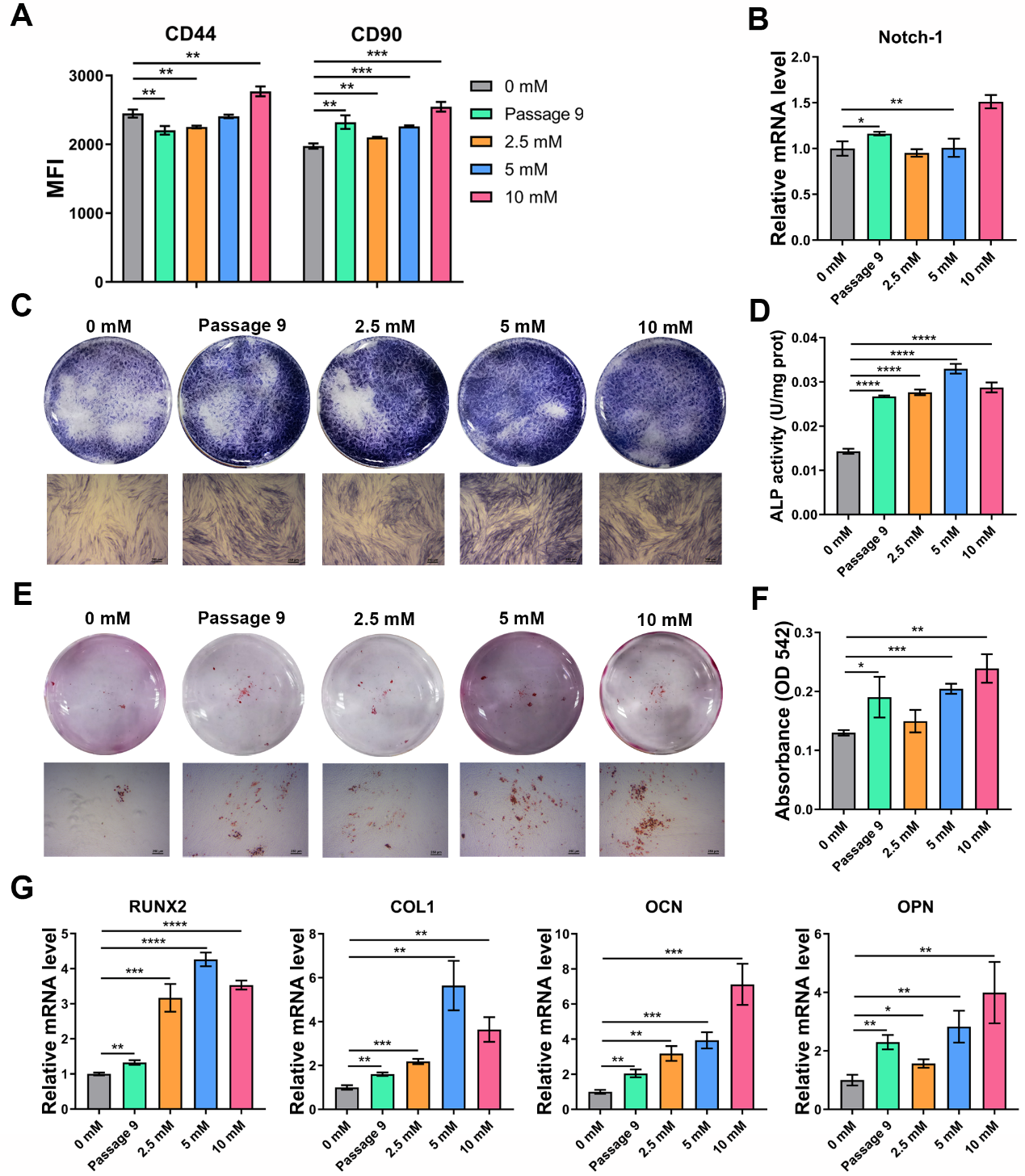


Supplementary Figure 4. Effects of various NAC concentrations on stem cell-specific markers and osteogenesis of hDFSCs at passage 20 (non-treated cells at passage 9 and 20 were used as the control). **(A)** MFI of CD44 and CD90 detected by flow cytometry. **(B)** Relative mRNA expression of Notch-1. **(C)** Photographs and micrographs depicting the osteogenic differentiation using ALP staining on day 5 after osteogenic induction. Scale bars: 250 μm. **(D)** Quantification of ALP activity. **(E)** Photographs and micrographs depicting the matrix mineralization using ARS staining on day 14 after osteogenic induction. Scale bars: 250 μm. **(F)** Semi-quantification of ARS staining. **(G)** Relative mRNA expression of osteogenic factors (RUNX2, COL1, OCN, OPN) after osteogenic culturing for 7 days. Statistically significant differences between groups were determined by p < 0.05 (*), p < 0.01 (**), p < 0.001 (***), p < 0.0001 (****).
