# Supplementary material for: N-acetylcysteine regulates dental follicle stem cell osteogenesis and alveolar bone repair via ROS scavenging
Source: Stem Cell Res Ther. 2022 Sep 8;13:466. doi: 10.1186/s13287-022-03161-y (PMC9461171; doi:10.1186/s13287-022-03161-y)
Supplement: Supplementary file 9 — Additional file 9. Table S4: GO enriched terms of biological process. [file 13287_2022_3161_MOESM9_ESM.doc]

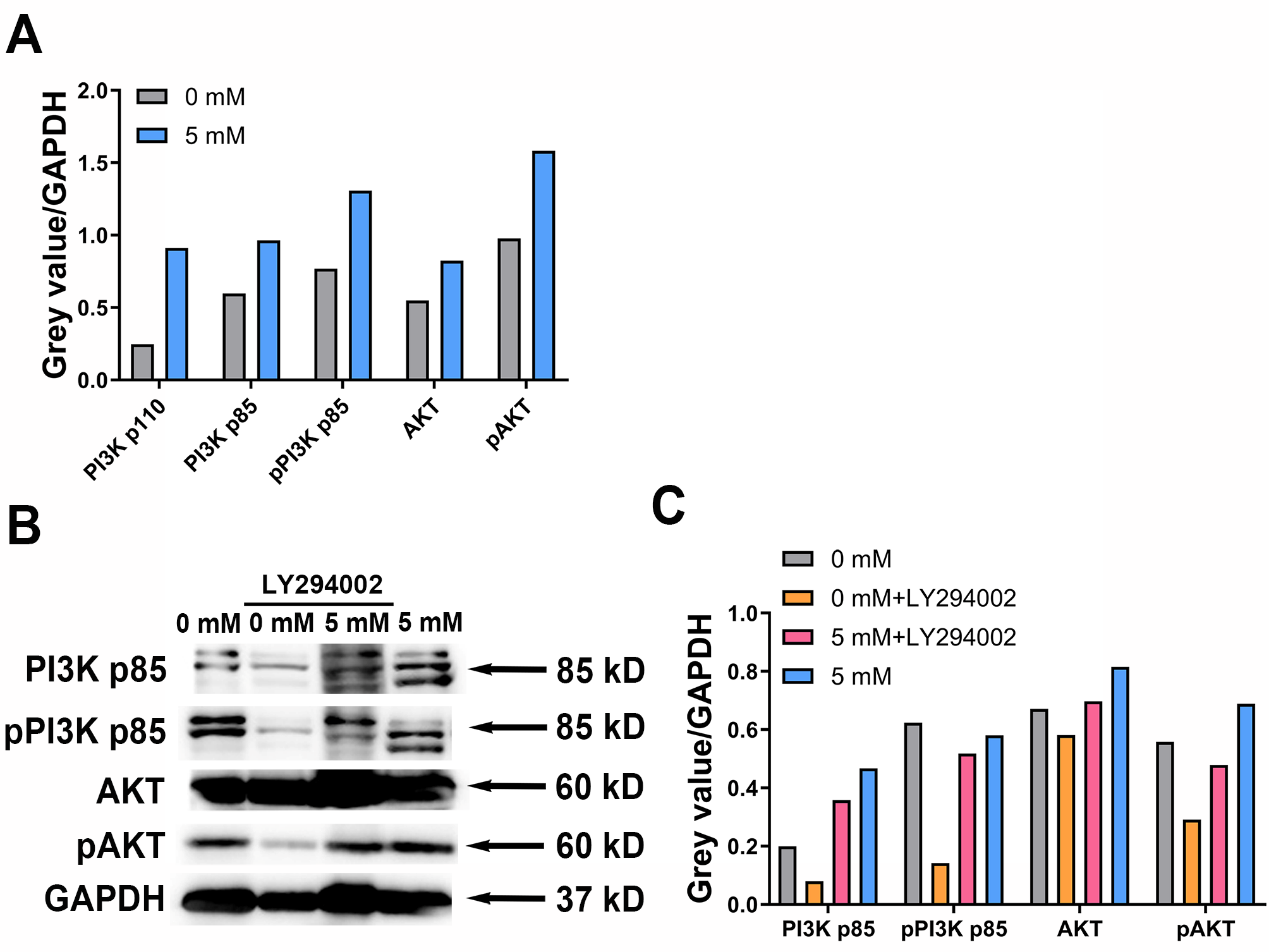


Supplementary Figure 5. Grayscale graph analysis of PI3K/AKT pathway proteins. **(A)** Grayscale graph analysis of PI3K/AKT pathway proteins after NAC treatment. **(B)** Representative western blots of PI3K/AKT pathway proteins after LY294002 treatment. **(C)** Grayscale graph analysis of PI3K/AKT pathway proteins after LY294002 treatment.
