# Supplementary material for: N-acetylcysteine regulates dental follicle stem cell osteogenesis and alveolar bone repair via ROS scavenging
Source: Stem Cell Res Ther. 2022 Sep 8;13:466. doi: 10.1186/s13287-022-03161-y (PMC9461171; doi:10.1186/s13287-022-03161-y)
Supplement: Supplementary file 10 — Additional file 10. Fig. S6: Tooth extraction socket wounds. (A) Photographs of tooth extraction socket wounds at day 7 post-extraction. (B) Wound areas analyzed by Image J. Statistically significant differences between groups were determined by P < 0.05 (*), P < 0.01 (**), P < 0.001 (***), P < 0.0001 (****). [file 13287_2022_3161_MOESM10_ESM.doc]

**Table S1.** Antibodies used in this study.

| **Type** | **Antibody** | **Catalogue number** | **Working**  **dilution** | **Company** |
| --- | --- | --- | --- | --- |
| Primary antibody | anti-GAPDH | YM3029 | 1:5000 | Immunoway Biotechnology (TX, USA) |
|  | anti-PI3K p85 | sc-374534 | 1:200 | Santa Cruz Biotechnology (CA, USA) |
|  | anti-PI3K p110 | sc-8010 | 1:400 | Santa Cruz Biotechnology (CA, USA) |
|  | anti-AKT | 4691T | 1:2000 | Cell Signaling Technology (MA, USA) |
|  | anti-phospho-PI3K p85 | 4228T | 1:1500 | Cell Signaling Technology (MA, USA) |
|  | anti-phospho-AKT | 4060T | 1:1500 | Cell Signaling Technology (MA, USA) |
| Secondary antibody | anti-mouse IgG | RS0001 | 1:2000 | Immunoway Biotechnology (TX, USA) |
|  | anti-rabbit IgG | RS0002 | 1:2000 | Immunoway Biotechnology (TX, USA) |
| Antibody for flow cytometry | FITC anti-human CD44 | 338803 | 1:20 | BioLegend (CA, USA) |
|  | PE anti-human CD90 | 328109 | 1:20 | BioLegend (CA, USA) |
|  | FITC Anti-Human CD29 | E-AB-F1049C | 1:20 | Elabscience (Wuhan, China) |
|  | APC Anti-Human CD31 | E-AB-F1050E | 1:20 | Elabscience (Wuhan, China) |
|  | PE Anti-Human CD117 | E-AB-F1150D | 1:20 | Elabscience (Wuhan, China) |
|  | FITC anti-rat CD11b/c | 201805 | 1:20 | BioLegend (CA, USA) |
|  | FITC anti-mouse/rat CD29 | 102205 | 1:20 | BioLegend (CA, USA) |
|  | PE Anti-Rat CD45 | E-AB-F1227D | 1:20 | Elabscience (Wuhan, China) |
|  | APC Anti-Rat CD90/Mouse CD90.1 | E-AB-F1226E | 1:20 | Elabscience (Wuhan, China) |
|  | PE anti-rat CD106 | 200403 | 1:20 | BioLegend (CA, USA) |
|  | FITC Mouse IgG1, κ Isotype Control | E-AB-F09792C | 1:20 | Elabscience (Wuhan, China) |
|  | PE Mouse IgG1, κ Isotype Control | E-AB-F09792D | 1:20 | Elabscience (Wuhan, China) |
|  | APC Mouse IgG1, κ Isotype Control | E-AB-F09792E | 1:20 | Elabscience (Wuhan, China) |
|  | FITC Mouse IgG2a, κ Isotype Control | E-AB-F09802C | 1:20 | Elabscience (Wuhan, China) |
|  | FITC Armenian Hamster IgG Isotype Control | E-AB-F09852C | 1:20 | Elabscience (Wuhan, China) |
